# Perioperative Treatment with Rivaroxaban and Dabigatran on Changes of Coagulation and Platelet Activation Biomarkers following Left Atrial Appendage Closure

**DOI:** 10.1155/2024/4405152

**Published:** 2024-03-12

**Authors:** Yao Yao, Yanli Li, Qinchun Jin, Xiaoye Li, Xiaochun Zhang, Qianzhou Lv

**Affiliations:** ^1^Department of Pharmacy, Zhongshan Hospital, Fudan University, Shanghai 200032, China; ^2^Department of Cardiology, Zhongshan Hospital, Fudan University, Shanghai 200032, China

## Abstract

Insufficient data exist regarding the investigation of the impact of novel oral anticoagulants (NOACs) on coagulation activation biomarkers in the context of left atrial appendage closure (LAAC) and device-related thrombosis (DRT). The study was designed to investigate the changes and presence of coagulation activation biomarkers between different antithrombotic strategies following LAAC. A total of 120 nonvalvular atrial fibrillation patients intolerant of long-term anticoagulants, who underwent successful WATCHMAN closure implantation, were enrolled (rivaroxaban, *n* = 82; dabigatran, *n* = 38). Blood samples were obtained from left atrium (LA) and left atrial appendage (LAA) during the operation and fasting blood samples on the same day of LAAC and 45 days after discharge. The biochemical indicators, thrombin-antithrombin complex (TAT), soluble P-selectin (sP-selectin), von Willebrand factor (vWF), and CD40 ligand (CD40L), were measured by enzyme-linked immunosorbent assay. The primary endpoints of this study were the efficacy and safety characteristics of different antithrombotic strategies, including DRT incidence, stroke or transient ischemic attack, systemic embolism, and clinical major and nonmajor bleeding complications during the follow-up of 180 days. The results revealed that TAT, vWF, sP-selectin, and CD40L levels in vein were significantly reduced by 2.4% (*p* = 0.043), 5.0% (*p* < 0.001), 8.7% (*p* < 0.001), and 2.5% (*p* = 0.043) from their baseline levels after rivaroxaban treatment. Conversely, no significant changes were detected in the dabigatran group. Furthermore, the plasma levels of platelet activation biomarkers (CD40L and sP-selectin) in both LA and LAA groups were significantly lower after anticoagulation with rivaroxaban, as compared to dabigatran treatment (CD40L: 554.62 ± 155.54 vs. 445.02 ± 130.04 for LA *p* = 0.0013, 578.51 ± 156.28 vs. 480.13 ± 164.37 for LAA *p* = 0.0052; sP-selectin: 2849.07 ± 846.69 vs. 2225.54 ± 799.96 for LA *p* = 0.0105, 2915.52 ± 1402.40 vs. 2203.41 ± 1061.67 for LAA *p* = 0.0022). Notably, the present study suggests that rivaroxaban may be more effective in the prevention of DRT for patients undergoing LAAC.

## 1. Introduction

Left atrial appendage (LAA) constitutes a critical of thrombosis in patients with atrial fibrillation (AF) [1]. More than 90% of thrombus in patients with nonvalvular atrial fibrillation (NVAF) originates from LAA, prompting the consideration of left atrial appendage closure (LAAC) as an alternative approach for stroke prevention in patients with NVAF [2, 3]. Long-term follow-up studies have indicated that LAAC intervention can mitigate the thrombotic risk associated with LAA thrombus formation, concurrently minimizing the bleeding risks associated with anticoagulant use [4, 5]. Notably, device-related thrombosis (DRT) after successful closure implantation on LAA poses a major challenge, with reported incidence ranging from 3% to 5%, associated with an increased risk of thrombus events [6, 7].

Despite current guidelines recommending early transient anticoagulation (45 days), determining the optimal antithrombotic strategy after LAAC requires a careful balance between thrombus (primarily DRT and thrombosis events) and bleeding prevention [8]. Importantly, limited comparative studies on biologic basis have been conducted between rivaroxaban (a selective FXa inhibitor) and dabigatran (a selective FIIa inhibitor).

Biomarkers such as CD40 ligand (CD40L) and soluble P-selectin (sP-selectin), closely related to platelet activation, along with thrombin antithrombin III (TAT) and vWF validated as the markers of coagulation activation, were utilized in this study [9–12]. Herein, we investigated these two categories of biomarkers to reflect the presence and variation of the changes in the biomarkers of platelet (CD40L and sP-selectin) and coagulation (vWF and TAT) activation under different anticoagulation regimens.

## 2. Methods

### 2.1. Study Population and Design

This prospective, observational, single-center study enrolled consecutive patients who underwent LAAC between January 2021 and December 2021. Ethics approval of antithrombotic protocols was granted by the Institutional Review Board at Zhongshan Hospital, Fudan University.

Patients eligible for WATCHMAN (Boston Scientific, Natick, MA, USA) implantation met the following inclusion criteria: (1) age > 18, (2) diagnosis as NVAF, (3) a potential ischemic stroke score (CHADS_2_) ≥ 2 or CHA_2_DS_2_-VAS_c_score ≥ 3, and (4) intolerant to long-term anticoagulants or at higher risk for bleeding. Participants who met the following criteria were excluded: (1) receiving long-term anticoagulants prior to closure implantation, (2) transfer to surgery due to LAAC procedure complications, and (3) planned AF ablation during the follow-ups. The study was not randomized, and the antithrombotic strategy was prescribed at the discretion of the operation physicians. Consecutive patients were categorized into two groups based on different anticoagulants, including dabigatran (110 mg bid, *n* = 38) and rivaroxaban (20 mg qd, *n* = 82).

### 2.2. Device Implantation Procedure and Perioperative Anticoagulation Strategy

All enrolled participants of this study received rivaroxaban or dabigatran for continuous four-week period to ensure a stable anticoagulation state before admission, after which they were admitted for the scheduled LAAC operation. During hospitalization, oral anticoagulants were ceased 24 hours before LAAC and resumed after closure implantation.

The detailed procedure for WATCHMAN device implantation has been previously described [13]. Considering patient characteristics and device type, the postimplant antithrombotic regimen could be individualized, ultimately left to the discretion of the physician. Patients received rivaroxaban or dabigatran (the approved anticoagulants at the time of the trial) at the manufacturer-recommended doses. A routine trans-esophageal echocardiography (TEE) examination was performed 45 days after device implantation to determine the presence of significant residual flow (>5 mm) or DRT. Anticoagulation was discontinued after confirming the absence of DRT and switched to dual antiplatelet therapy (DAPT), including aspirin (100 mg qd) and clopidogrel (75 mg qd), until 6 months. Subsequently, long-term aspirin therapy was maintained.

### 2.3. In-Hospital and Out-of-Hospital Follow-Ups

Detailed demographic and baseline clinical features were extracted from hospital information systems (HIS). Each patient's risk for potential thromboembolism and bleeding was assessed using the CHA_2_DS_2_-VAS_c_ and HAS-BLED scores, and laboratory parameters related to the liver, renal function, and coagulation were systematically recorded. TEE was conducted during in-hospital management to rule out cardiac effusion.

Routine outpatient follow-ups for each enrolled participant comprised up to three repeated TEE examinations scheduled approximately at 45 days, 180 days, and annually postprocedure to assess the absence of residual leak and DRT.

### 2.4. Blood Sampling and Laboratory Parameters

A 5F pigtail catheter was inserted through trans-septal sheath to obtain blood samples from the left atrium (LA) and LAA during the operation. Fasting blood samples were obtained on the same day as the LAAC procedure and 45 days after discharge. Each blood sample was meticulously collected into vacutainer tubes prefilled with 0.5 mL of 3.2% buffered sodium citrate (Becton Dickinson). Subsequently, the samples were centrifuged at 3000 rpm for 25 min at room temperature and then stored at -80°C until batch analysis. Centrifugation was initiated within 30 min of sample collection.

Biochemical indicators were measured by the Department of Laboratory Medicine of Zhongshan Hospital, Fudan University. The levels of TAT, sP-selectin, vWF, and CD40L in the supernatants from blood samples were detected using enzyme-linked immunosorbent assay (ELISA) kits (YaJi Biological, Shanghai, China) according to the manufacturer's instructions, with the detection ranges of 2-100 ng/mL, 10-1000 ng/L, 20-500 U/L, and 30-2400 ng/L, respectively.

### 2.5. Clinical Outcomes

Concerning the differences in the risks associated with each antithrombotic regimen, the primary endpoints evaluated the efficacy and safety characteristics of each strategy during the follow-up of 180 days, including (1) the incidence of DRT, defined as a well-circumscribed and uniformly echo-dense mass lying on the LA side of the device, measured by TEEs, (2) stroke or transient ischemic attack (TIA) diagnosed by neurology examination based on computed tomography (CT) or magnetic resonance imaging (MRI), (3) systemic embolism (SE) in addition to stroke and TIA, and (4) clinical major and nonmajor bleeding complication definitions according to the guidance of the International Society on Thrombosis and Haemostasis [14]. The secondary endpoints are aimed at comparing biochemical indicators, including TAT, sP-selectin, vWF, and CD40L, between different anticoagulation strategies.

### 2.6. Statistical Analyses

Previous data showed that changes in platelet and coagulation activation biomarkers following anticoagulants were associated with a 20-30% decrease for LAAC patients. Conservatively, estimating a post-treatment difference in platelet and coagulation activation biomarkers of about 25% between the two groups, with a standard deviation of no more than 30%, a sample size of 32 patients per group (assuming a dropout rate of 10%) should provide adequate power (class II error rate *β* = 0.1) to detect a significant difference (*α* = 0.05) in platelet and coagulation activation biomarker levels.

Continuous variables were presented as mean ± standard deviation (SD). Categorical variables were expressed with frequencies or percentages *n* (%). Baseline characteristics were compared between each group using *t* tests and *χ*^2^ tests/Fisher's precision probability test, as appropriate. The comparable variables included platelet and coagulation biomarkers, namely, TAT, sP-selectin, vWF, and CD40L levels, for each eligible participant throughout the study. The occurrence of DRT or thrombosis was analyzed to examine differences in clinical outcomes between the two groups. Kaplan-Meier curves were constructed to display/show time-to-first DRT or system thrombosis, and comparisons were made using log-rank tests for trend and the Cox regression analysis.

A *p* value of 0.05 was considered to be the threshold for statistical significance. Statistical analysis was performed using SPSS software (version 22.0; IBM, Armonk, USA).

## 3. Results

### 3.1. Baseline Characteristics

A total of 120 patients intolerant to long-term anticoagulants and undergoing LAAC were finally enrolled in this study.  Table 1 presents a comprehensive overview of the main baseline and procedural characteristics for the study population. No significant differences were observed in terms of age, gender, predetermined stroke, and bleeding risk factors among the two investigated groups. The proportions of individuals with high thromboembolic risk, as indicated by a CHA_2_DS_2_-VAS_c_ score of >5, were 12.5% and 5% for rivaroxaban and dabigatran, respectively, without statistical significance. Regarding bleeding risks, as determined by a HAS-BLED score of ≥3, 76.3% and 97.5% of cases were observed for rivaroxaban and dabigatran, respectively, without significant differences. The baseline characteristics were well-matched across all other variables.

### 3.2. Clinical Efficacy and Safety Evaluation

**Figure 1:**
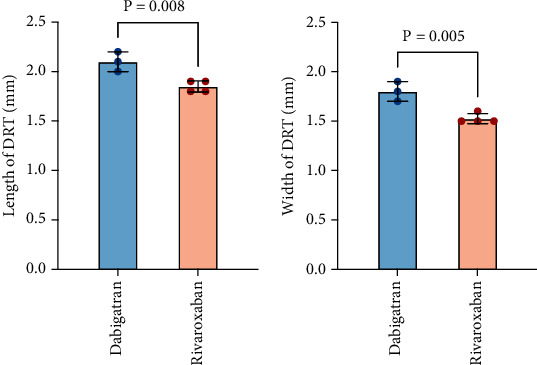
Length and width of device-related thrombus (DRT) evaluated with transesophageal echocardiography.

The early-phase formation of DRT was investigated, as reflected by the incidence of DRT and thrombus size. DRTs were detected in both anticoagulant groups following TEE imaging within 180 days. Specifically, in the rivaroxaban group, DRT occurred in 4 of 82 patients (4.8%), while in the dabigatran group, it was observed in 3 of 38 patients (7.9%). Complete thrombus resolution in rivaroxaban and dabigatran was recorded after switching to warfarin (INR 2-3). The median length and width of DRT in the rivaroxaban group were 1.85 (1.80-1.90) mm and 1.52 (1.50-1.60) mm, significantly lower than those in the dabigatran (length = 2.10 (2.01-2.22) mm, *p* = 0.008; width =1.80 (1.70-1.90) mm, *p* = 0.004) (Figure 1).

**Figure 2:**
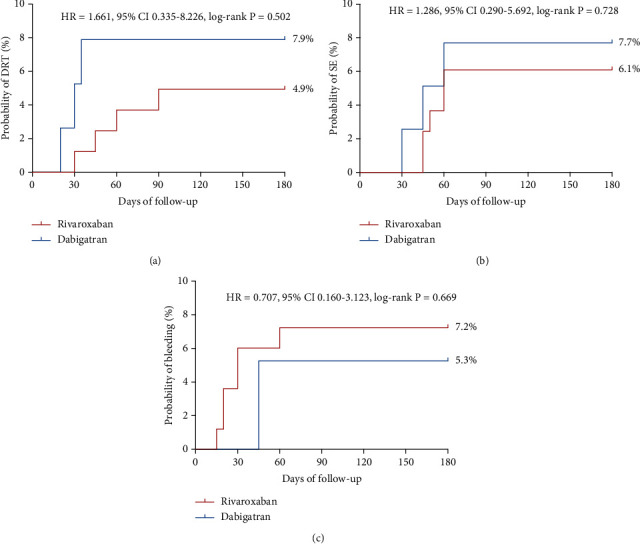
Kaplan-Meier survival curves for device-related thrombus (DRT), TE events, bleeding and rehospitalization according to different anticoagulants: (a) the Kaplan-Meier survival curve of DRT, (b) systemic embolism (SE) events, and (c) bleeding events.

Furthermore, the KM survival curve in Figure 2 indicated that patients in the dabigatran group were more likely to experience a shorter time to DRT compared to those in the rivaroxaban group (HR = 1.661, 95% CI 0.335-8.226, log-rank *p* = 0.502) (Figure 2(a)). No significant difference was noted in terms of TE events between the two groups (Figure 2(b)).

The evaluation of anticoagulation-related bleeding complications is illustrated in Table 2. Cumulative incidences of bleeding complications, including gastrointestinal hemorrhage, operation site hemorrhage, and skin ecchymosis during anticoagulation therapy, were similar in both the dabigatran and rivaroxaban groups (*p* > 0.05) (Figure 2(c)). There was no significant difference between the two groups with respect to key hematological parameters, including the Hb and PLT levels under the bleeding threshold (*p* > 0.05) (Table 2).

### 3.3. Coagulation and Platelet Activation Biomarkers of Thrombus Indication in Vein, LA, and LAA

**Figure 3:**
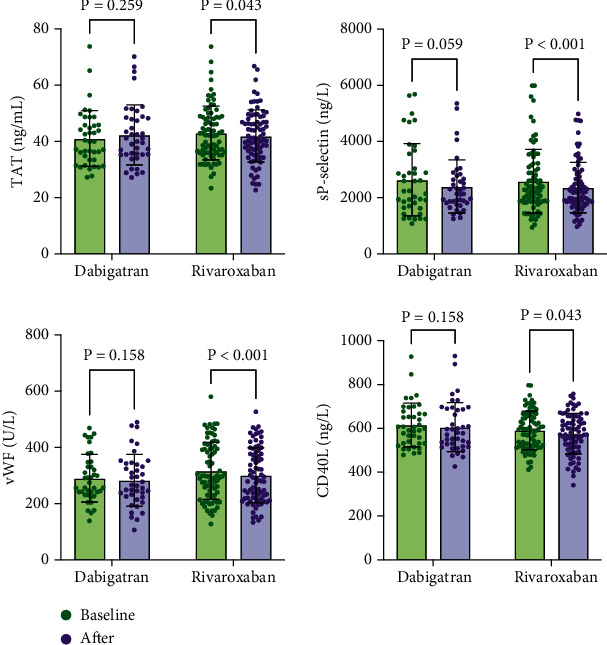
Changes of coagulation and platelet activation biomarkers in vein.

Figure 3 illustrates the measurements of platelet (assessed by CD40L and sP-selectin) and coagulation (assessed by TAT and vWF) system activation in vein. The mean baseline levels of TAT, vWF, sP-selectin, and CD40L were similar between the dabigatran and rivaroxaban groups (43.03 ± 9.92 vs. 47.25 ± 12.96, *p* = 0.078; 301.83 ± 86.84 vs. 335.77 ± 127.76, *p* = 0.090; 2646.44 ± 1286.87 vs. 2535.64 ± 1160.52, *p* = 0.635; and 636.05 ± 99.04 vs. 664.77 ± 122.67, *p* = 0.202, respectively). A significant decrease was found in platelet and coagulation system activation after rivaroxaban treatment (*p* < 0.05 for these biomarkers), while no significant changes were detected in the dabigatran group. Notably, TAT and vWF levels in the rivaroxaban group were lower by 2.4% (SE: 0.43, *p* = 0.043) and 5.0% (SE: 4.29, *p* < 0.001) than baseline, respectively. Similarly, sP-selectin and CD40L levels in the rivaroxaban group remained 8.7% (SE: 63.43, *p* < 0.001) and 2.5% (SE: 6.29, *p* = 0.043) lower than baseline.

**Figure 4:**
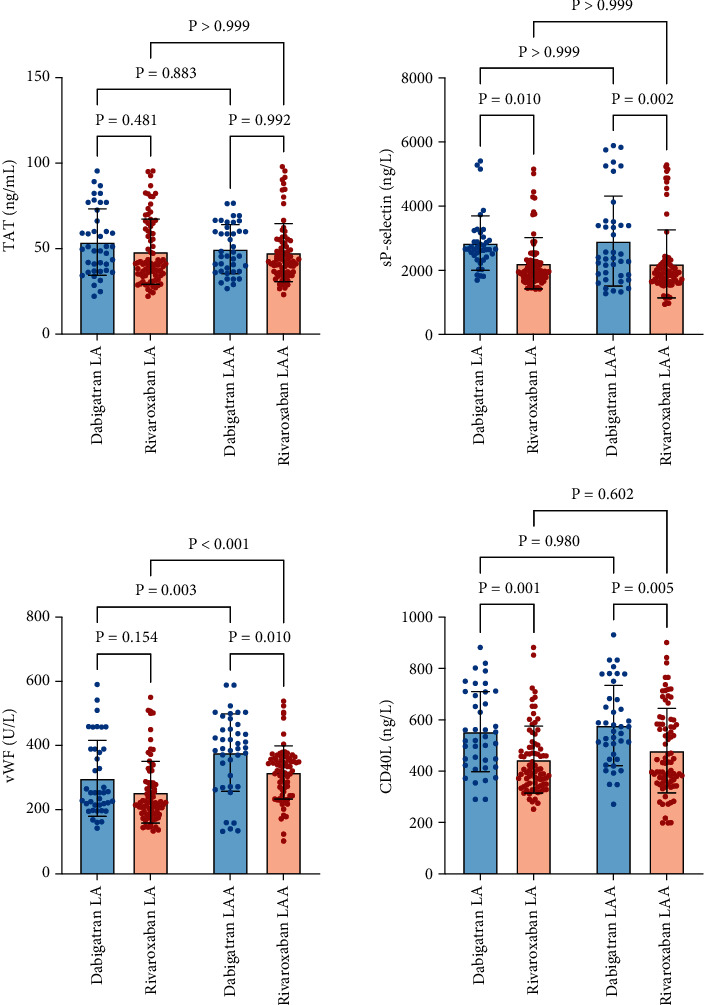
The presence of coagulation and platelet activation biomarkers in LA and LAA.

Figure 4 presents the comparison of soluble coagulation and platelet activation markers in LA and LAA. The plasma levels of platelet activation biomarkers (CD40L and sP-selectin) in the LA and LAA groups were significantly lower after anticoagulation with rivaroxaban compared to dabigatran treatment (554.62 ± 155.54 versus 445.02 ± 130.04 for LA *p* = 0.0013 and 578.51 ± 156.28 versus 480.13 ± 164.37 for LAA *p* = 0.0052, CD40L; 2849.07 ± 846.69 versus 2225.54 ± 799.96 for LA *p* = 0.0105 and 2915.52 ± 1402.40 versus 2203.41 ± 1061.67 for LAA *p* = 0.0022, sP-selectin).

The plasma levels of the coagulation activation biomarkers (TAT) showed no significant difference between two groups in LA and LAA (53.87 ± 19.40 versus 48.25 ± 19.09, *p* = 0.4809 and 49.75 ± 14.32 versus 47.69 ± 16.98, *p* = 0.9916). Meanwhile, the plasma level of vWF was markedly reduced after rivaroxaban medication in LAA (378.08 ± 118.59 versus 316.27 ± 96.39, *p* = 0.0104), while no significant difference was exhibited in LA (297.73 ± 121.01 versus 254.45 ± 83.05, *p* = 0.1538).

The plasma levels of vWF in LAA were significantly higher than those in LA (*p* < 0.05), while CD40L, sP-selectin, and TAT levels exhibited no significant difference between the LAA and LA (*p* > 0.05).

### 3.4. Risk Factor Analysis for Elevated D-Dimer

Among the 120 enrolled patients, 22.5% (27/120) participants presented with elevated D-dimer levels greater than 0.5 mg/mL, indicating a high thromboembolism risk. Multivariate logistic regression was performed to identify the independent risk factor for elevated D-dimer, including age, CHA_2_DS_2_-VAS_c_ score, high-sensitivity C-reactive protein (hs-CRP), coagulation factors, plasma platelet and coagulation activation biomarkers, and anticoagulants. In the combined patient analysis, univariable analysis was initially performed to identify the potential risk factors for elevated D-dimer. Afterwards, multivariable analysis illustrated that hs-CRP (OR = 1.154, 95% CI: 1.036-1.286, *p* = 0.009) and higher CD40L level in LAA (OR = 1.004, 95% CI: 1.000-1.007, *p* = 0.049) were independent predictors for elevated D-dimmer, as illustrated in Table 3.

## 4. Discussion

This study prospectively investigated the changes of coagulation and platelet activation biomarkers following LAAC operations with different anticoagulants. The main findings of the study are as follows. First, anticoagulation with rivaroxaban resulted in a significant reduction of coagulation and platelet activation biomarkers in vein as compared to dabigatran. Second, rivaroxaban demonstrated the ability to decrease the levels of coagulation and platelet activation biomarkers in LA and LAA. Third, patients on long-term dabigatran exhibited a higher incidence of SE and rehospitalization. Current anticoagulation regimens have demonstrated efficacy in preventing thrombus formation after successful LAAC implantation by promoting the complete endothelialization of occluders [15]. While pharmacological clinical studies have shown the superiority of anticoagulation strategies over antiplatelet therapy, the optimal management of anticoagulants remains uncertain due to the lack of direct comparisons between different anticoagulants. Herein, this prospective study is presented to contribute insights into the optimal anticoagulation regimen post-LAAC operation.

In real-world patients undergoing LAAC with the WATCHMAN device, DRT is relatively rare, and there is limited research directly comparing DRT rates among patients using different NOACs. Our study significantly contributes to the expanding body of evidence on antithrombotic strategies in the context of LAAC, focusing on their impact on DRT and thromboembolic prevention. Specifically, we explored the feasibility and safety of NOACs as an alternative to warfarin for preventing DRT, which aligns with previous research findings [16].

Our results indicate that early rivaroxaban anticoagulation following LAAC may be more protective for reducing DRT incidence compared to dabigatran, although no statistical significance was found (rivaroxaban 4.8% vs. dabigatran 7.9% at 6 months). These findings align with previous research highlighting the potential benefits of NOACs, particularly the anti-Xa inhibitor rivaroxaban, in preventing DRT. This advantage may be attributed to their ability to attenuate platelet activity and aggregation by inhibiting coagulation factor Xa [17]. Moreover, the EWOLUTION study, which investigated various anticoagulation regimens following WATCHMAN LAAC, reported that the NOAC group had the lowest incidence of thrombus formation on the device surface [18]. These results suggest that NOACs, known for their shorter half-life, effectively prevent thrombus formation in the low-flow area of the LA. Real-world evidence from the EWOLUTION registry further supports the use of NOACs as adjuvant therapy post-WATCHMAN implantation, reinforcing our study's findings [18]. In addition, the ADRIFT study demonstrated lower thrombin generation with reduced rivaroxaban doses as an alternative to dual antiplatelet therapy (DAPT) post-LAAC, underscoring the potential of NOACs in DRT prevention [19]. While prior studies have identified potential predictors of DRT, such as a history of TIA or stroke, permanent atrial fibrillation, vascular disease, left atrial appendage diameter, and left ventricular ejection fraction [20], recent data from the EWOLUTION real-world registry challenge this conventional wisdom. These latest findings suggest that the presence of DRT may not be linked to an increased risk of stroke or SE [7].

In terms of primary endpoints related to stroke or SE events, our study did not reveal a significant difference between the two anticoagulation strategies. However, it is noteworthy that patients medicated with rivaroxaban exhibited lower rates of SE when compared to those taking dabigatran, in line with previous research findings [21]. To provide context, the ROCKET-AF trial reported a stroke or SE incidence of 1.7% per year [22]. The LAAOS III trial demonstrated the effectiveness of a novel approach that combines LAAC with NOACs versus NOACs alone, resulting in a significant reduction in the composite outcome of ischemic stroke, TIA, and SE in the LAAC group (hazard ratio: 0.67) [23]. It is worth noting that discontinuing DOAC therapy can increase the risk of subsequent cardioembolic events [24]. Therefore, short-term post-LAAC use of NOAC medications may help improve patient compliance and, consequently, reduce the risk of cardioembolic events [25].

Considering both antithrombotic efficacy and safety, particularly with regard to bleeding complications, our results revealed no significant differences between the two NOAC groups in terms of laboratory biomarkers such as hemoglobin and platelet count (*p* > 0.05). These findings are in line with a comparative study suggesting a similar safety profile between dabigatran and rivaroxaban [26]. While LAAC inherently reduces the risk of bleeding compared to long-term oral anticoagulants for patients with NVAF, the challenge remains in selecting the most suitable antithrombotic strategy, especially for individuals intolerant to prolonged anticoagulation and at high risk of stroke [27]. Several studies have explored the feasibility and safety of DOACs following LAAC, indicating that DOACs may offer a beneficial solution in preventing bleeding events among Chinese individuals [28]. In particular, Asian populations tend to favor lower DOAC doses due to their lower average body mass index and distinct bleeding susceptibility [29, 30].

Similar to any cardiovascular intervention procedure, thrombosis complication after LAAC was closely associated with coagulation and platelet activation that peaked on the seventh day postoperation [31]. Our present study indicates that rivaroxaban is more effective in reducing platelet activation in vein, suggesting a reduction in thrombin generation on the closure of surface. Our finding is consistent with reported studies showing platelet inhibition with rivaroxaban medication [32, 33]. From a pathophysiological perspective, platelet activation and aggregation are directly mediated via FXa signaling pathways during thrombus formation, which may be attenuated under rivaroxaban medication [34]. Conversely, elevated platelet reactivity is determined in dabigatran therapy, mainly by enhancing the thrombin receptor density on platelet [35]. These in vivo observations have been recently corroborated by prospective clinical studies, providing further insights into coagulation changes after a successful LAAC operation with different antithrombotic regimens [21, 34]. One previous study illustrated that rivaroxaban might potentially prevent periprocedural DRT and thrombus events as compared to dabigatran [18]. The prothrombotic status was measured through platelet aggregation biomarkers in terms of sP-selectin and CD40L, which showed a significant reduction level in the rivaroxaban group.

To the best of our knowledge, there is insufficient data available regarding the investigation of the impact of NOACs on coagulation and platelet activation biomarkers in the context of LAAC and DRT. The enhanced thrombin generation in LA and LAA might be the main cause of DRT, attributed to fibrin deposition on device surface [36]. Before the LAAC operation, all participants received rivaroxaban or dabigatran for a continuous four weeks to ensure a steady drug concentration [37]. We observed no significant differences in the presence of coagulation activation biomarkers in LA and LAA between two groups, while a significant tendency toward lower degree of platelet activation biomarkers was found after rivaroxaban medication. The results of present study, indicating a significant reduction of platelet activation biomarkers in LA and LAA after rivaroxaban medication, support the recommendation of short anticoagulation with rivaroxaban after a successful LAAC. A previous clinical study demonstrated a trend of reduction in sP-selectin and CD40L levels within 180 days following LAAC after receiving anticoagulants [38]. This mild platelet activation inhibition on coagulation system might explain some cases of late DRT after LAAC among patients taking dabigatran.

Some limitations of the present study are acknowledged. The primary limitation of this observation study is the nature of nonrandomized and controlled trial, which makes it difficult to obtain an accurate conclusion. Consequently, external validation data are essential to corroborate the findings, and future randomized controlled trials (RCTs) may provide more definitive evidence. Additionally, the relatively low frequency of TEE monitoring could have resulted in a reduced DRT confirmation. To establish the clinical efficacy and safety among different antithrombotic strategies more robustly, larger sample sizes and increased monitoring may be necessary. Owing to the low incidence of DRT and the relatively short duration of follow-up, we did not thoroughly assess the association between DRT and coagulation activation biomarkers. Furthermore, this study exclusively enrolled participants receiving NOACs, specifically rivaroxaban and dabigatran; caution should be exercised when attempting to extrapolate the findings to the other NOACs, as differences may exist in the performance of these medications. Drawing direct comparisons of clinical effectiveness between dabigatran and rivaroxaban with coagulation and platelet activation biomarkers can be considered indirect and potentially unreliable. Further research, ideally in the form of head-to-head RCTs, may provide more definitive evidence for comparing these anticoagulant strategies. The disproportionate distribution of patients between the rivaroxaban and dabigatran groups might introduce bias and weaken the comparative analysis. Cohort studies or RCTs with more balanced patient cohorts are needed to address this limitation. Lastly, the utilization of biomarkers for predicting thrombosis complications introduces a level of indirection and may be considered less reliable compared to direct diagnostic methods. While we have included information about the use of biomarkers in this study, it is important to recognize that this method may not be as robust or conclusive as direct diagnostic tests. The potential for systematic bias due to the indirect nature of biomarkers should be noted, and future studies may benefit from a comparative analysis with more direct diagnostic approaches. The absence of follow-up data on coagulation and platelet activation biomarkers is another limitation of this study. Further studies should consider collecting and analyzing such data to confirm the association between these biomarkers and clinical outcomes more comprehensively.

## 5. Conclusion

Our study found that rivaroxaban exerted a more potential antiplatelet effect by decreasing platelet activation biomarkers, in addition to its well-known potent anticoagulatory capacity, compared to dabigatran. This may contribute to a reduced frequency of DRT and improved outcome in patients following LAAC. However, due to the limited samples and disproportionate distribution, further clinical studies are required to establish the clinical efficacy and safety among different antithrombotic strategies for post-LAAC.

## Figures and Tables

**Table 1:** Baseline and procedural characteristic comparisons of the study population.

Baseline characteristics	Rivaroxaban (*n* = 82)	Dabigatran (*n* = 38)	*p* value
Age (y)	68.81 ± 9.49	69.40 ± 11.12	0.764
Gender (male, %)	40 (48.8)	23 (60.5)	0.231
Stroke and bleeding risk factors			
CHF	3 (3.7)	0 (0.0)	0.551
Hypertension	16 (19.5)	8 (21.1)	0.844
Age > 75 y	20 (24.4)	11 (28.9)	0.596
Diabetes mellitus	52 (63.4)	24 (63.2)	0.978
Stroke/TIA	56 (68.3)	23 (60.5)	0.404
Vascular disease	33 (40.2)	21 (55.3)	0.124
Age (65-74 y)	38 (46.3)	18 (47.4)	0.916
Sex category (female gender)	40 (48.8)	17 (44.7)	0.680
Abnormal renal function	19 (23.2)	6 (15.8)	0.354
Abnormal liver function	6 (7.3)	3 (7.9)	1.000
Bleeding history	7 (8.5)	4 (10.5)	0.741
Drug interactions	42 (51.2)	21 (55.3)	0.680
Alcohol	8 (9.8)	2 (5.3)	0.501
Coronary heart disease	13 (15.9)	8 (21.1)	0.486
WATCHMAN size (dm, cm)	29.03 ± 1.84	28.88 ± 1.76	0.669
LVEF, %	62.21 ± 6.53	62.88 ± 4.40	0.564
CHA_2_DS_2_-VAS_c_ score			
3-4	44 (53.7)	20 (52.6)	0.916
≥5	10 (12.2)	2 (5.3)	0.335
HAS-BLED score			
0-2	20 (24.4)	11 (28.9)	0.596
≥3	62 (75.6)	27 (71.1)	0.596

Values are mean ± SD, *n* (%). *p* value was calculated with two-way ANOVA analysis and represented with interaction, *p* < 0.05 was considered significant. CHF: congestive heart failure; TIA: transient ischemic attack; LVEF; left ventricular ejection fraction; CHA_2_DS_2_-VAS_c_ risk calculated with risk factors congestive heart failure, hypertension, age ≥ 75 years, diabetes mellitus, prior stroke or transient ischemic attack or thromboembolism, vascular disease, age 65-74 years, and sex category; HAS-BLED risk score calculated with hypertension, abnormal renal or liver function, stroke, bleeding, labile international normalized ratio, elderly, drugs, or alcohol.

**Table 2:** Anticoagulation complication comparison with NOACs for LAAC.

	Rivaroxaban (*n* = 82)	Dabigatran (*n* = 38)	*p* value	OR (95% CI)
Bleeding complications				
Gastrointestinal hemorrhage (%)	4 (4.9%)	2 (5.3%)	0.928	0.927 (0.177-4.842)
Operation site hemorrhage (%)	2 (2.4%)	1 (2.6%)	0.950	0.927 (0.087-9.909)
Skin ecchymosis (%)	3 (3.7%)	2 (5.3%)	0.682	0.695 (0.121-3.990)
Laboratory parameters				
PLT < 125 (%)	7 (8.5%)	3 (7.9%)	0.906	1.081 (0.296-3.954)
Male: Hb < 120 (%)	4 (4.9%)	1 (2.6%)	0.567	1.854 (0.214-16.030)
Female: Hb < 110 (%)				

OR: odds ratio; CI: confidence interval; PLT: platelet count; Hb: hemoglobin.

**Table 3:** Logistic proportional hazard regression analyses for elevated D-dimer.

Variables	Univariate analysis		Multivariate analysis	
	OR (95% CI)	*p* value	OR (95% CI)	*p* value
Age	1.073 (1.020-1.129)	0.007	1.068 (0.991-1.150)	0.084
Gender (male)	0.542 (0.227-1.294)	0.168		
BMI (kg/m^2^)	1.138 (0.930-1.394)	0.210		
CHA_2_DS_2_-VAS_c_ score	1.176 (0.830-1.665)	0.362		
Hypertension	3.873 (0.849-17.667)	0.080	6.739 (0.727-62.444)	0.093
Diabetes mellitus	1.021 (0.420-2.480)	0.964		
Hyperlipidemia	1.182 (0.387-3.612)	0.769		
Stroke	0.953 (0.385-2.362)	0.917		
Anticoagulation protocols (rivaroxaban vs. dabigatran)	3.632 (1.160-11.364)	0.027	0.023 (0.245-4.270)	0.975
Closure size	0.881 (0.689-1.127)	0.313		
LVDD	1.041 (0.969-1.118)	0.271		
LVDS	1.041 (0.963-1.125)	0.310		
LVEF	0.961 (0.905-1.019)	0.184		
hs-CRP	1.164 (1.053-1.287)	0.003	1.154 (1.036-1.286)	0.009
Coagulation and platelet activation biomarkers				
TAT				
LA	1.015 (0.993-1.037)	0.178		
LAA	1.007 (0.982-1.034)	0.583		
Vein	0.993 (0.962-1.026)	0.677		
sP-selectin				
LA	1.000 (1.000-1.001)	0.038	1.001 (1.000-1.001)	0.071
LAA	1.000 (1.000-1.001)	0.277		
Vein	1.000 (1.000-1.000)	0.954		
vWF				
LA	1.002 (0.998-1.005)	0.397		
LAA	1.001 (0.997-1.006)	0.555		
Vein	1.000 (0.997-1.004)	0.933		
CD40L				
LA	1.000 (0.997-1.003)	0.928		
LAA	1.002 (1.000-1.005)	0.072	1.004 (1.000-1.007)	0.049
Vein	1.002 (0.998-1.006)	0.271		

The hazard ratio (HR) and the 95% confidence interval (CI) were calculated using univariate Cox regression, and variables with a statistical significance of *p* < 0.10 were entered into the multivariate stepwise Cox proportional hazard regression model. BMI: body mass index; sCr: serum creatinine; RAASi: renin angiotensin aldosterone system inhibitor; LVDD; left ventricular end diastolic dimension; LVDS: left ventricular end systolic diameter; LVEF: left ventricular ejection fraction; hs-CRP: high-sensitivity C-reactive protein.

## Data Availability

The datasets used and analyzed in this current study are available from the corresponding authors upon reasonable request.
